# The Critical Care Pain Observation Tool for Families (CPOT-Fam)

**DOI:** 10.1080/24740527.2026.2708710

**Published:** 2026-09-24

**Authors:** Anmol Shahid, Bonnie G. Sept, Corson Johnstone, Victoria S. Owen, Stephana J. Moss, Rebecca Brundin-Mather, Karla D. Krewulak, Andrea Soo, Jeanna Parsons Leigh, Céline Gélinas, Kirsten M. Fiest, Henry T. Stelfox

**Affiliations:** a Faculty of Nursing, University of Calgary, Calgary, Canada; b Department of Critical Care Medicine, Cumming School of Medicine, University of Calgary & Alberta Health Services, Calgary, Alberta, Canada; c School of Health Administration, Faculty of Health, Dalhousie University, Halifax, Nova Scotia, Canada; d Ingram School of Nursing, McGill University, and Centre for Nursing Research and Lady Davis Institute, Jewish General Hospital - CIUSSS West-Central Montreal, Montreal, Quebec, Canada; e Department of Psychiatry and Hotchkiss Brain Institute, and the Department of Community Health Sciences, Cumming School of Medicine, University of Calgary, Calgary, Alberta, Canada; f O’Brien Institute for Public Health, Cumming School of Medicine, University of Calgary, Calgary, Alberta, Canada; g Faculty of Medicine and Dentistry, University of Alberta, Edmonton, Alberta, Canada

**Keywords:** Pain, family engagement, patient-centered care, family-centered care, critical care, intensive care unit

## Abstract

**Background:**

Patients in the intensive care unit (ICU) are often unable to communicate. Family caregivers could assist in pain assessment using a family-adapted version of the Critical Care Pain Observation Tool (CPOT-Fam).

**Aims:**

We aimed to evaluate the feasibility, acceptability, and convergent validity (compared with nurses’ CPOT scores) of the CPOT-Fam for use by family caregivers to assess pain behaviors in ICU patients.

**Methods:**

We collected simultaneous pain assessments from family caregivers (CPOT-Fam) and from nurses (CPOT) during periods of rest and standard care procedures in ICU patients. Feasibility was assessed by both the proportion of family caregivers who consented to participate and who completed the CPOT-Fam. Acceptability was assessed using surveys of the sentiments of family caregivers and nurses toward family involvement in pain assessment. Convergent validity was evaluated using Spearman’s rank correlation between CPOT-Fam and CPOT scores.

**Results:**

Fifty-two family caregivers were approached to participate in this study, 43 (82.3%) consented, and all 43 (100%) completed the study. Most family caregivers (90.0%) and nurses (81.4%) reported that families have a valuable role in pain assessment in the ICU. Correlation between CPOT-Fam and CPOT scores (43 paired assessments; [95% confidence intervals]) at rest was ρ = 0.419 [0.127, 0.644], and during standard care procedures (n = 12 paired assessments) was ρ = 0.743 [0.276, 0.926].

**Conclusions:**

The CPOT-Fam appears to be a feasible and acceptable tool for family caregivers to assess pain in ICU patients. Clinical evaluation of CPOT-Fam reliability and validity is warranted.

## RÉSUMÉ

**Contexte :** Les patients aux soins intensifs sont souvent incapables de communiquer. Les proches aidants pourraient contribuer à l’évaluation de leur douleur à l’aide d’une version de l’Outil d’observation de la douleur aux soins intensifs adaptée à la famille (CPOT-Fam).

**Objectifs :** Cette étude visait à évaluer la faisabilité, l’acceptabilité et la validité convergente du CPOT-Fam (déterminée par comparaison avec les scores CPOT attribués par les infirmières) lorsqu’il est utilisé par les proches aidants pour évaluer les comportements liés à la douleur chez les patients aux soins intensifs.

**Méthodes :** Nous avons recueilli des évaluations concomitantes de la douleur auprès de proches aidants (CPOT-Fam) et d’infirmières (CPOT) pendant les périodes de repos et les interventions de soins courants chez des patients aux soins intensifs. La faisabilité a été évaluée à la fois par la proportion de proches aidants ayant consenti à participer à l’étude et par la proportion de ceux ayant rempli le CPOT-Fam. L’acceptabilité a été évaluée au moyen d’enquêtes portant sur les points de vue des proches aidants et des infirmières à l’égard de la participation des proches à l’évaluation de la douleur. La validité convergente a été évaluée au moyen du coefficient de corrélation des rangs de Spearman entre les scores du CPOT-Fam et ceux du CPOT.

**Résultats :** Cinquante-deux proches aidants ont été invités à participer à cette étude; 43 (82,3 %) y ont consenti et les 43 (100 %) ont terminé l’étude. La plupart des proches aidants (90,0 %) et des infirmières (81,4 %) ont indiqué que la contribution des familles à l’évaluation de la douleur aux soins intensifs était précieuse. Au repos, la corrélation entre les scores du CPOT-Fam et ceux du CPOT, calculée à partir de 43 évaluations appariées, était de ρ = 0,419 (IC à 95 % : [0,127; 0,644]). Pendant les interventions de soins courants, elle était de ρ = 0,743 (IC à 95 % : [0,276; 0,926]) pour 12 évaluations appariées.

**Conclusions :** Le CPOT-Fam semble être un outil utilisable et acceptable par les proches aidants pour évaluer la douleur chez les patients aux soins intensifs. Sa fidélité et sa validité devraient maintenant faire l’objet d’une évaluation clinique.

## Introduction

Family caregivers (e.g., family members, friends) can recognize behaviors signifying the presence or severity of pain in their loved ones.^1,2^ This ability can be valuable in the intensive care unit (ICU), where many patients are unable to self-report pain due to critical illness.^3–6^ Shortcomings in pain assessment may affect patient experience and outcomes (e.g., intensity of pain, duration of mechanical ventilation, length of stay in ICU).^7^ Studies have shown that pain evaluations provided by their family caregivers can be closely aligned with patients’ self-reported pain ratings.^1,8^ This suggests that family caregivers may have an important role in the pain assessment of ICU patients.

Bedside ICU nurses commonly use the Critical Care Pain Observation Tool (CPOT) to assess pain in patients incapable of self-reporting their pain in the ICU.^9–11^ Data suggests that nurse CPOT scores for non-communicative critically ill patients correlate with patient heart rate and blood pressure and are higher during painful procedures.^12^ Given the widespread use of the CPOT, alongside growing recognition of the potential for family caregivers to contribute to pain assessment in patients unable to self-report, our research team adapted the CPOT to create a family-oriented pain assessment tool (CPOT-Fam). Patient and family research partners, knowledge users, and academic researchers jointly co-adapted the tool by simplifying CPOT terminology, adding illustrations to represent scoring options, and conducting iterative evaluations and revisions.^13^ During preliminary testing of the CPOT-Fam in the ICU setting, family caregivers reported predominantly positive experiences, describing the tool as straightforward and easy to use without formal training.^14^

To further our work in creating a family-oriented pain assessment tool,^13–15^ we tested the CPOT-Fam in ICU settings to determine:

1. the feasibility of recruiting family caregivers to use the CPOT-Fam to assess pain (measured as the proportion of family caregivers approached for study participation who consented to participate; >80% defined as feasible);
2. the feasibility of CPOT-Fam use by family caregivers (measured as the proportion of family caregivers participants who completed the CPOT-Fam; >80% defined as feasible);
3. the acceptability of family caregivers using the CPOT-Fam (measured by survey responses of family caregiver and nurse sentiments toward family involvement in pain assessment); and
4. the convergent validity of the CPOT-Fam, determined by comparing pain behavior scoring between family caregivers and bedside nurses.

## Methods

The University of Calgary Conjoint Health Research Ethics Board (REB 21–0748) provided ethics approval for the conduct of the study. Alberta Health Services (the health custodian for the study site) provided approval to approach family caregivers of ICU patients for study participation and to access health data. Permission to adapt the CPOT for family caregiver use was granted by the American Association of Critical Care Nurses (AACN).^8^

### Design

We used a cross-sectional study to assess the feasibility and acceptability of collecting pain scores (CPOT-Fam and CPOT) from family caregivers for critically ill patients who were unable to communicate. Simultaneous assessments of pain by family caregivers using the CPOT-Fam and nurses using the CPOT were used to assess convergent validity.

### Study setting

We conducted this study in the 36-bed medical-surgical ICU at the Foothills Medical Center in Calgary between October 2022 and July 2023. The Foothills Medical Center is the primary tertiary care center serving Southern Alberta.

### Participants

Family caregivers were eligible for inclusion if they were: 1) 18+ years old, 2) able to communicate in English (i.e., understand, read, speak), and 3) able to provide informed written consent and surrogate consent on behalf of the patient. Family caregivers were identified with assistance from beside nurses, who had knowledge of the individuals involved in the day-to-day care of their patients.

### Recruitment

Family caregivers were approached based on their availability and appropriateness for participation in the study, as determined in collaboration with bedside nurses. In some cases, family caregivers were not available at the time of data collection. In others, nurses advised against approaching families due to circumstances. Bedside nurses provided verbal consent to be part of the study.

### Procedures

When both family caregivers and research team members were present in the ICU, family caregivers were asked to complete the paper-based CPOT-Fam,^14^ while the patient was at rest and during a standard care procedure (e.g., suctioning, repositioning). Family caregivers were also asked to complete paper-based surveys collecting: 1) their demographic information (age, sex, level of education, primary language spoken at home, and relationship to the critically ill patient) and 2) their sentiments regarding pain assessment of their loved one in the ICU (open-ended responses). Research team members (AS, BGS, RP) were available in-person to answer questions from family caregivers to facilitate participation.

Simultaneously but independently from the family caregivers, bedside nurses provided CPOT scores and completed a survey on their perceptions of the family role in ICU pain assessment. CPOT scores during standard care procedures were only collected from family caregivers and nurses when the timing of the procedure coincided with study procedures (i.e., the patient needed to be suctioned or repositioned as a part of their care). Family caregiver and bedside nurse pain assessments were collected independently and were masked to one another. eCritical, a population-based provincial critical care clinical information system which captures demographic, clinical, and outcomes data for all admitted ICU patients, was used to collect patient information including the patient’s age, sex, reason for the patient’s admission to ICU, and ICU length of stay.^15^

### Instruments

#### Critical Care Pain Observation Tool (CPOT)

The CPOT can be used to assess pain through four behavioral indicators: facial expression, body movements, compliance with ventilation if intubated, or vocalization if not intubated, and muscle tension. Each item is scored from 0 (lowest) to 2 (highest), yielding a maximum cumulative score of 8 to reflect pain behavior intensity.^16,17^ A threshold score of ≥2 is indicative of pain requiring pain management.^18^

The CPOT is used around the world and is available in multiple languages (English,^19^ French, Dutch,^20^ Finnish,^21^ German,^22^ Italian,^23^ Korean,^24^ Mandarin,^25^ Polish,^26^ Portuguese,^27^ Spanish,^28^ Turkish^29^). To date, the CPOT has been validated in several subpopulations of adult critically ill patients (brain-injured/neurosurgery,^16,30^ cardiac surgery,^31,32^ delirious,^33^ palliative^34^) and pediatric patients^35^ (orthopedic surgery^36^).

#### Critical Care Pain Observation Tool for Families (CPOT-Fam)

The CPOT-Fam, like the CPOT, assesses pain using four behavioral indicators: facial expression, body movements, compliance with ventilation (if intubated) or vocalization (if not intubated), and muscle tension. Each item is scored from 0 (lowest) to 2 (highest), yielding a maximum cumulative score of 8 to reflect pain behavior intensity. The CPOT-Fam was specifically created by our team and collaborators for use by family caregivers without the need for formal training. The methodology used to adapt the CPOT for family caregiver use has been described previously.^13–15^

### Surveys

#### For family caregivers

Family caregivers were asked to complete a demographic survey that captured their age group, sex, highest level of education, ethnic or cultural background, language spoken at home, place of residence, household income, and relationship to the patient in the ICU. In addition, family caregivers were asked to respond to a series of closed- and open-ended questions. Open-ended questions were designed to provide participants with the opportunity to describe their experiences and sentiments. Questions assessed comfort in identifying pain in their family member (“*How comfortable do you feel in your ability to tell whether your family member is experiencing pain?,”* with response options ranging from *very comfortable* to very *uncomfortable*), perceptions of the role of families in pain assessment *(“What do you feel is the role of families in pain assessment”)*, and assessments of empowerment to act on identified pain (“*If you identify pain in your family member, do you feel empowered to act on this information?*”). Family caregivers who indicated that they did not feel empowered were invited to provide an open-ended explanation.

#### For bedside nurses

Nurses were asked a single open-ended survey question *(“What do you feel is the role of families in pain assessment?”)* and were invited to elaborate to the level of detail they chose.

### Operational and logistic considerations

Research team members documented operational and logistic considerations related to study recruitment and data collection. These included identifying when family members were most likely to be present and willing to participate in the study, when healthcare providers were most receptive to being approached, and how to coordinate data collection during standard care procedures.

### Statistical analysis

Quantitative data were analyzed using SPSS version 29.0 (IBM, Chicago, USA). Participant enrollment, characteristics and completion of study procedures were summarized using frequencies and percentages. The CPOT-Fam and CPOT median scores were presented as [(Interquartile range, IQR) and minimum (min) and maximum (max) scores]. To address the objective of evaluating convergent validity, Spearman’s rank-order correlation coefficient (ρ) was used to examine the association between pain scores reported by family caregivers using the CPOT-Fam and those reported by bedside nurses using the standard CPOT for each patient.^37^ Correlations were calculated for overall CPOT-Fam and CPOT scores, as well as for individual behavioral domains of the tools, including facial expression, body movements, ventilator compliance (for intubated patients) or vocalization (for non-intubated patients), and muscle tension. Correlation coefficients were interpreted according to established guidelines, where values of 0–0.3 indicate negligible correlation, 0.3–0.5 low correlation, 0.5–0.7 moderate correlation, 0.7–0.9 high correlation, and 0.9–1.0 very high correlation.^38^

### Content analysis

Responses to the open-ended question posed to families and bedside nurses were reviewed in-duplicate using NVIVO 12 (QSR International, Burlington, MA, USA).^39^ Comments were classified as either having “positive,” “neutral,” or “negative” sentiment toward family involvement in pain assessment (simplified, manual version of sentiment analysis).^40^ Two research team members (AS, BGS) compared their independent and masked (to each other’s evaluations) interpretation of sentiments and discussed converging and diverging opinions.

### Sample size and power considerations

We aimed for a sample size of 40, with which we could estimate the proportion of approached family caregivers who consent to participate in the study of 80% to within a 95% confidence interval of ±12.4%. This sample size was selected as per available recommendations and usage in previous work in critical care settings to determine the feasibility and acceptability of the CPOT-Fam and identify any operational and logistic considerations needed to conduct a larger study.^41,42^

## Results

**Figure 1.**
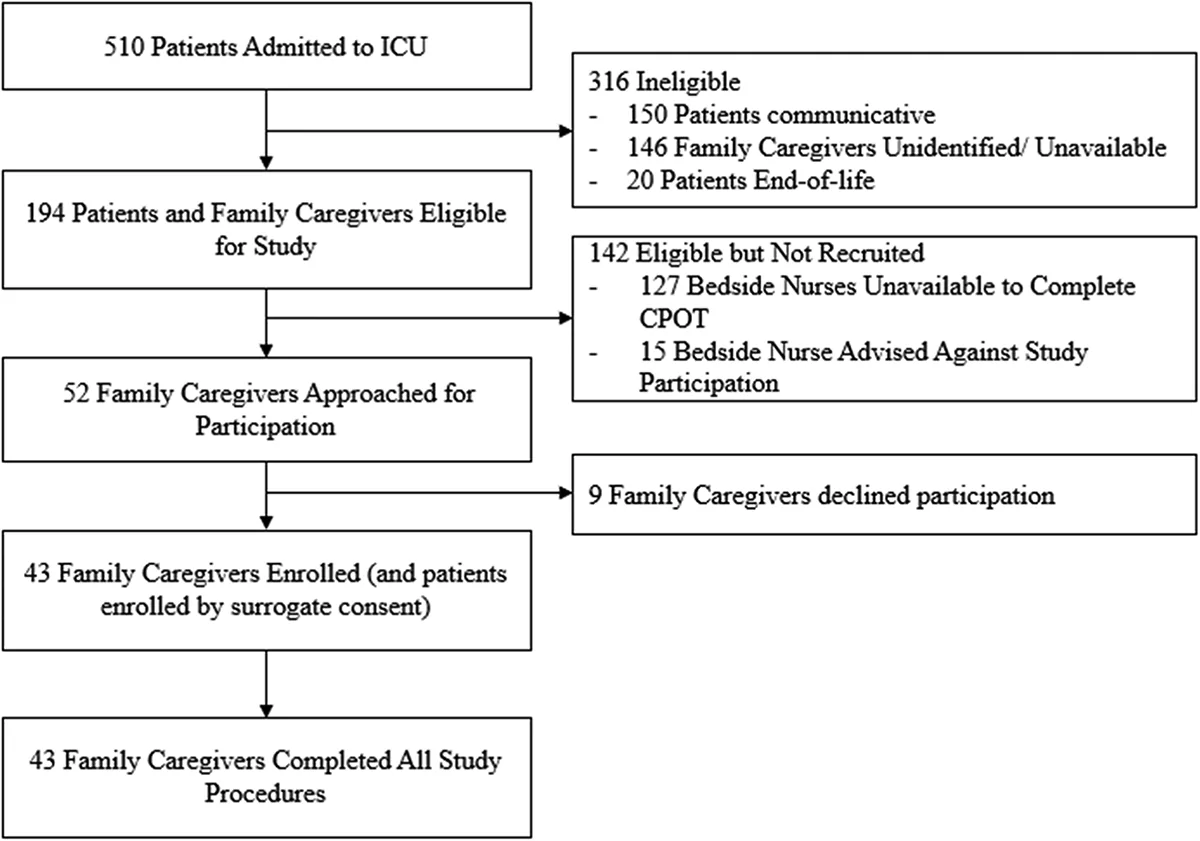
Flow diagram showing participant selection. **Note**: ICU = intensive care unit, CPOT = Critical Care Pain Observation Tool.

During the study recruitment period (Oct 2022–July 2023), 510 patients were admitted to the study ICU. Of these, 194 satisfied the inclusion criteria and 52 family caregivers of eligible patients were approached to participate in the study, of which 43 (82.7%, 95% CI 72.4%–93.0%) consented to participate. All individuals who consented to participate completed (n = 43; 100%) participation in the study (Figure 1). Most family caregivers were female (n = 28; 65.1%), belonged to a primarily English-speaking household (n = 28, 65.1%), and were college or university educated (n = 32; 74.4%). Fourteen (32.5%) family caregivers were spouses and 24 (56.0%) were first-degree relatives of the ICU patient. Of the patients enrolled, most were 40+ years of age (n = 31; 72.1%), male (n = 28; 65.1%), and admitted to ICU for a medical (i.e., non-surgical) reason (n = 35; 81.4%). Participant characteristics are detailed in Table 1.

**Table 1.** Characteristics of study participants (n = 43).

Characteristics n(%)	Patients (n = 43)	Family caregivers (n = 43)
**Age (years)**		
18–39	12 (27.9%)	8 (18.6%)
40–59	13 (30.2%)	11 (25.6%)
60+	18 (42.0%)	24 (55.8%)
**Female**	15 (34.9%)	28 (65.1%)
**Reason for ICU admission**		
Medical	35 (81.4%)	
Surgical	8 (18.6%)	
**ICU length of stay median, days [IQR]**	4 [8.1]	
**Education**		
High school diploma or less		11 (26.0%)
Some college or university		26 (60.5%)
Graduate or professional degree		6 (14.0%)
**Primary language spoken at home**		
English		28 (65.1%)
Other		15 (31.3%)
**Relationship with ICU patient**		
Significant other		14 (32.5%)
First degree relative (parent, child, sibling)		24 (56.0%)
Other (distance relative, friend)		5 (11.6%)

### Family caregiver survey

#### Closed-ended questions

All (n = 43, 100%) family caregiver participants provided responses to the closed-ended survey questions. In response to “*How comfortable do you feel in your ability to tell whether your family member is experiencing pain?,”* thirty-seven (86.0%) family caregivers indicated they were “very comfortable” or “moderately comfortable,” four (9.3%) indicated they were “not comfortable or uncomfortable” and two (4.7%) indicated being “moderately uncomfortable.” All participants (n = 43; 100.0%) indicated that they would feel empowered to act if they identified pain in their family caregiver.

#### Open-ended questions

Nine of 43 (20.9%) family caregivers answered the question *“What do you feel is the role of families in pain assessment?”* with open text responses. The responses were categorized into frequencies of positive, neutral, and negative sentiments (some comments included multiple sentiments). Most responses reflected positive sentiments by family members toward their role in pain assessment, citing “intimate knowledge,” and willingness to “advocate” for the patient. There was one instance of a family caregiver being “unsure” that they had a role in pain assessment in the ICU. Select quotes from family caregivers are shown below.

I want to see him [the patient] as comfortable as possible.

I can tell [about pain] from her movement – been with her everyday and know her well.

#### Bedside nurse survey (open-ended)

They [family] also know patient prior to admission hence they have a better understanding of pain tolerance level. They can be a good patient advocate at the bedside.

Forty-two of 43 nurses responded to the question *“What do you feel is the role of families in pain assessment?”* As these responses were more detailed, they were analyzed and categorized into subcategories reflecting positive, neutral, negative, and multiple sentiments regarding the role of families in pain assessment. In total, 89 instances of nurses expressing sentiments falling into the aforementioned categories were identified. A majority (n = 57 instances; 64.0%) of responses favored having a role for families in pain assessment (Table 3), citing benefit to families, patients, and clinical decision-making. Many nurses felt that the positive aspects of families having a role in pain assessment can be attributed to their intimate knowledge of the patient (n = 24 instances; 26.9%), ability to notice acute changes in patient pain (n = 10 instances; 11.2%) and facilitating communication between clinicians and patients. Some nurses provided statements indicating that family caregiver participation in pain assessment in the ICU could have negative effects because families may not be able to assess pain accurately (n = 14 instances; 15.7%), could be easily distressed by witnessing pain in the ICU (n = 8 instances; 9.0%), and might create controversy between families and healthcare providers on how to manage the pain (n = 3 instances; 3.4%). Select quotes from bedside nurses are shown below.

Families often underestimate or overestimate pain. I take family opinion into consideration. The upside is that they know the patient. The downside could be the wrong family member could have bad intentions - we may not know the history between family or how close they are with the patient.

Families are often helpful in assessment of pain because they can understand and perceive the patient’s signs of pain better than us [nurses]. Patients may also feel more comfortable sharing their pain with families.

### CPOT-fam correlation with CPOT

#### Patient at rest (N = 43 paired assessments)

We collected 43 paired assessments while the patient was at rest. In these assessments, the CPOT-Fam and CPOT median scores [(Interquartile range, IQR) and minimum (min) and maximum (max) scores] at rest were 0.00 [IQR (0.00–2.00), min = 0, max = 8] and 1.00 [(0.00–1.00), min = 0, max = 6], respectively (Figure 2a). Correlation (ρ), [95% confidence intervals] between total CPOT-Fam and CPOT scores was ρ = 0.419, [0.127, 0.644], 0.005. Correlation was highest for the *body movements (*ρ = 0.558, [0.302, 0.739], <0.001) item and lowest for the *facial expression* item (ρ = 0.068, 0.663 [−0.245, 0.369]). Correlations and percent agreement by each behavioral item are shown in Table 2.

**Table 3.** Summary of bedside nurse open-text responses to “What do you feel is the role of families in pain assessment?,” reported as number of instances (% of total n = 89 instances).

Sentiment	Sub-category of sentiment	Instances (%)
*Positive*	Family role in pain assessment benefits patient	34 (38.2%)
	Family role in pain assessment benefits family (e.g., enhances empowerment, engagement)	5 (5.60%)
	Family role in pain assessment improves clinician decision making	18 (20.2%)
*Neutral*	Family role in pain assessment is sometimes appropriate	7 (7.87%)
*Negative*	Family role in pain assessment could negatively affect patient	17 (19.1%)
	Family role in pain assessment could negatively affect family	5 (5.60%)
	Family role in pain assessment could negatively affect clinical decision making	3 (3.37%)

**Figure 2a.**
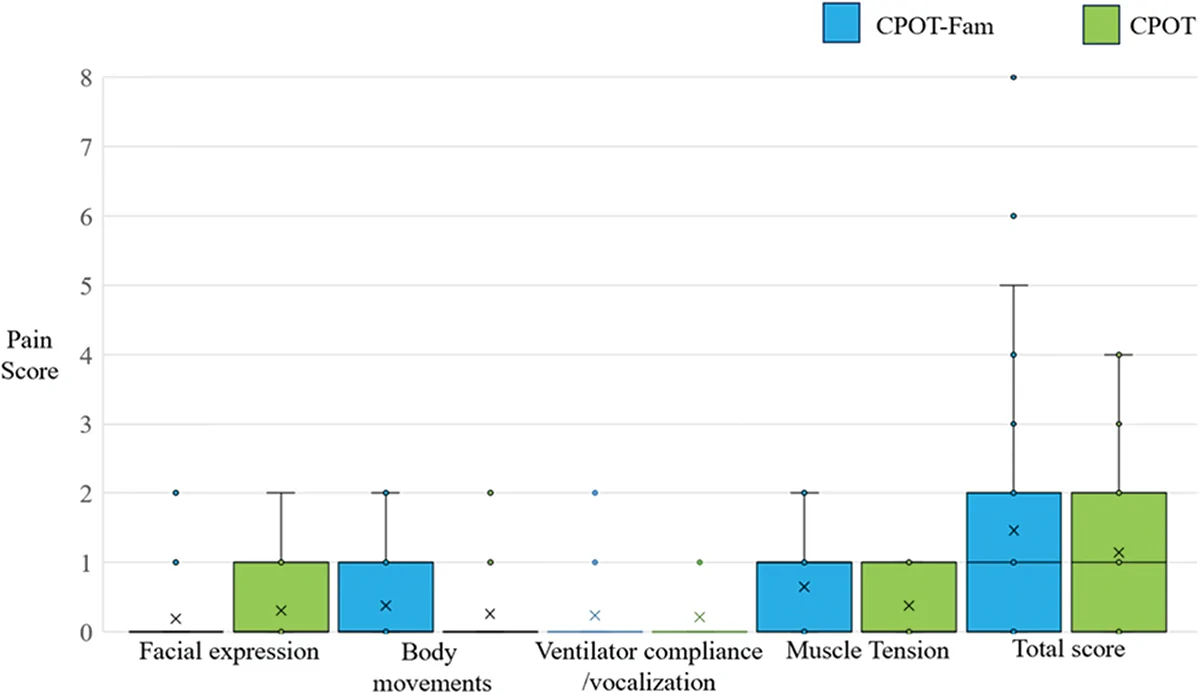
Distribution of simultaneous pain scores by families (CPOT-Fam) and nurses (CPOT) when the patient is at rest.

**Note**: X presents the data mean; lines between boxes represent median scores; outliers are shown; Note 2: If the difference between quartile 1 and 3 values is negligible for a given category, a box is not shown in the figure (for example, ventilator compliance/vocalization).

**Table 2.** Correlation (Spearman’s rank order correlation coefficient **(ρ)**), and crude percent agreement between family caregiver (CPOT-Fam) and bedside nurse (CPOT) pain scores at rest and during standard care procedures.

	AT REST (n = 43)		DURING STANDARD CARE PROCEDURE (n = 12)	
	% exact score agreement^a^	Correlation (ρ), [95% confidence intervals]^a^	% agreement	Correlation (ρ) [95% confidence intervals]^a^
Total score (0–8)	34.9%	0.419 [0.127,0.644]	25.0%	0.743[0.276,0.926]
Ventilator compliance /vocalization (score 0–2)	74.4%	0.313 [0.005, 0.567]	58.3%	0.398[−0.246, 0.798]
Facial expression (score 0–2)	55.8%	0.068 [−0.245, 0.369]	50.0%	0.714, [0.219,0.917]
Body movements (score 0–2)	74.4%	0.558 [0.302, 0.739]	33.3%	0.763[0.412, 1.00]
Muscle tension (score 0–2)	60.4%	0.514 [0.245, 0.710]	58.3%	0.573, [0.021, 0.868]

^a^Percent agreement and Spearman’s rank order coefficient **(ρ)**, [95% confidence interval], p-value for behavioral items of pain assessment were generated through comparison of pain scores between family caregivers (CPOT-Fam) and nurses (CPOT) for a given patient.

#### Patient during standard care procedure (N = 12 paired assessments)

We collected 12 pain assessments during standard care procedures that may cause pain. In these assessments, the CPOT-Fam and CPOT median scores [(IQR) and (min) and (max) scores] were: 2.50 [(2.00–7.00), min = 0, max = 8] and 1.00 [(0.00–3.50), min = 0, max = 8], respectively (Figure 2b). Median CPOT-Fam scores were higher than CPOT scores for every behavioral item. Correlation (ρ), [95% confidence intervals], p-value between total CPOT-Fam and CPOT scores was 0.743, 0.006 [0.276, 0.926]. Correlation was highest for the *body movements (*ρ *= *0.763, 0.002 [0.412, 1.00]) item and lowest for the *ventilator compliance/vocalization (*ρ = 0.398,0.200[−0.246, 0.798]) items (Table 2).

**Figure 2b.**
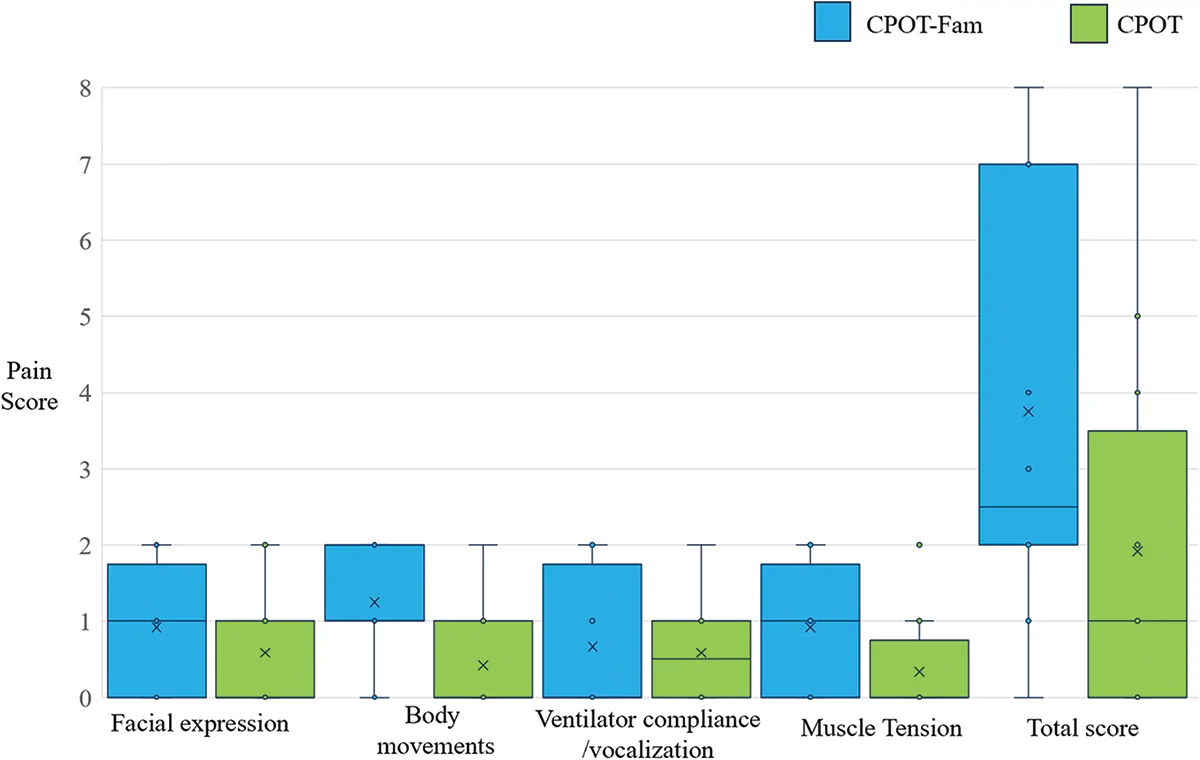
Distribution of simultaneous pain scores by families (CPOT-Fam) and bedside nurses (CPOT) during a standard care procedure (i.e., suctioning or repositioning).

**Note**: X presents the data mean; lines between boxes represent median scores; outliers are shown.

## Discussion

The CPOT-Fam was co-developed by ICU patients, family caregivers, nurses and physicians to help family caregivers participate in pain assessment.^13,14^ In this study, we found that it was feasible to both recruit family caregivers to participate in the study and to use the CPOT-Fam. Most caregivers and nurses reported positive sentiments regarding family caregiver involvement in pain assessment. Family caregiver pain scores (CPOT-Fam) and bedside nurse pain scores (CPOT) demonstrated low correlation when patients were at rest (ρ = 0.419) and moderate correlation during standard care procedures (ρ = 0.743), suggesting that agreement between assessors may be context dependent. We also identified that complex ICU workflows complicate the coordination of paired family caregiver–nurse pain assessments, particularly around standard care procedures.

In non-ICU environments, research has strongly indicated that family caregivers are well-acquainted with individualized pain behaviors of patients and could play a significant role in pain assessment and management.^43^ Although few studies have examined family caregiver pain assessment in ICU settings where patients are unable to communicate, select evidence suggests greater concordance between patient self-reported pain scores and family caregiver assessments (ICC = 0.43) than with bedside nurse assessments (ICC = 0.29).^43,44^ Other studies have demonstrated that family caregivers can accurately assess pain in critically ill patients a majority of the time (73.5%), particularly when higher levels of pain are present.^3,45^ Although we were only able to collect 12 paired (CPOT-Fam/CPOT) scores during standard care procedures, our findings appear consistent with this literature. We found that family caregiver and bedside nurse pain scores may be more highly correlated during standard care procedures than periods of rest, suggesting that family caregivers may be especially attuned to observable pain-related behaviors during potentially painful events.

While family caregivers could be valuable in appraising pain for ICU patients who cannot self-report their pain, previous studies of clinician attitudes have underscored that family caregiver factors, relationship dynamics, communication capabilities, and health literacy can notably impede family caregiver involvement in patient care in the ICU.^46^ Of note, there have been previously published reports about families overestimating pain^45^ and clinicians underestimating depending on workload and responsibilities.^3^ In our study, we observed family caregivers to provide higher pain scores than nurses. However, survey data collected in our study showed that while the aforementioned factors are notable, bedside nurse sentiments toward family caregiver involvement in pain assessment are largely positive. Literature in this area also shows greater agreement between patient pain scores and family caregiver pain scores.^5^ Together, these findings may reflect shifting sentiments among bedside clinicians toward greater family caregiver engagement in the ICU, a practice supported by contemporary clinical practice guidelines.^47^ This is consistent with the growing literature demonstrating the feasibility and value of family caregiver engagement in other aspects of ICU care, including delirium assessment and participation in liberation from mechanical ventilation.^2,48^

### Limitations

Considering that bedside ICU nurses continue to experience burnout as a longer-term consequence of the COVID-19 pandemic,^49^ this study was designed to minimize interruption to nurse workflow. Pain scores during standard care procedures were only obtained if these procedures were already taking place. This resulted in a smaller sample size for pain measurements during standard care procedures highlighting the need to interpret these scores with caution.^50^ Our study does not provide any information on the reliability of the CPOT-Fam. Multiple factors (e.g., tool reliability, operator training/experience, responsiveness to changing clinical circumstances) could potentially explain the observed wider distribution of CPOT-Fam scores in our study, and this warrants exploration in future studies. Furthermore, as patients in this study were not able to self-report their pain (the CPOT is used in the ICU for patients unable to self-report their pain, and the CPOT-Fam has been designed to be used by family caregivers), there was no reference standard for pain (i.e., the patient’s self-report). Consequently, we cannot determine how closely family caregiver and bedside nurse pain assessments were aligned with the patient’s pain experience. Future studies will need to consider evaluating the criterion validity of the CPOT-Fam by having critically ill patients self report their own pain (the reference standard). We also recognize that sex- and gender-based differences may influence the perception and assessment of pain.^51^ These considerations may be particularly relevant when there appear to be differences between the sex of patients (more likely to be male) and family caregivers (more likely to be female).^2,3^ Future studies should incorporate sex- and gender-based analyses, including between-group analyses to explore whether pain scores differ according to patient and caregiver sex and gender.

### Considerations for designing future studies

Informal observations collected during our study can be used to inform the design of a future, prospective, multi-center study to evaluate the reliability and validity of the CPOT-Fam. These observations highlight the need to 1) standardize the timing of CPOT completion by nurses to facilitate simultaneous completion of pain assessments with family caregivers, 2) develop closer collaboration with unit staff, allowing the research team to have a greater awareness of when standard care procedures are being planned for a given patient and when family caregivers will be present, and 3) plan study activities for times when family caregivers may be the most available and likely to participate in the study (often just after rounds, or evenings and weekends).

## Conclusions

Testing of the CPOT-Fam, a proxy pain assessment tool for family caregiver of ICU patients, shows the tool is feasible and acceptable for family caregiver use in the ICU. Co-developed with patient advocates, family caregivers, and healthcare providers, the CPOT-Fam has the potential to engage and empower family caregivers in the ICU and to improve pain assessment. Clinical evaluation of CPOT-Fam reliability and validity is warranted.

## References

[1] Vanderbyl BL, Gelinas C. Family perspectives of traumatically brain-injured patient pain behaviors in the intensive care unit. Pain Manag Nurs. 2017;18(4):202–12. doi:10.1016/j.pmn.2017.04.005. 28601477

[2] Fiest KM, Krewulak KD, Ely EW, et al. Partnering with family members to detect delirium in critically ill patients. Crit Care Med. 2020;48(7):954–961. doi:10.1097/CCM.0000000000004367. 32332281

[3] Puntillo KA, Neuhaus J, Arai S, Paul SM, Gropper MA, Cohen NH, Miaskowski C. Challenge of assessing symptoms in seriously ill intensive care unit patients: can proxy reporters help? Crit Care Med. 2012;40(10):2760–2767. doi:10.1097/CCM.0b013e31825b94d8. PMC3712644 22890258

[4] Gupta M, Sahi MS, Bhargava AK, Talwar V. The prevalence and characteristics of pain in critically Ill cancer patients: a prospective nonrandomized observational study. Indian J Palliat Care Sep-Dec. 2015;21(3):262–267. doi:10.4103/0973-1075.164894. PMC4617031 26600692

[5] Gelinas C. Pain assessment in the critically ill adult: recent evidence and new trends. Intensive Crit Care Nurs. 2016;34:1–11. doi:10.1016/j.iccn.2016.03.001. 27067745

[6] Herr K, Coyne PJ, Ely E, Gelinas C, Manworren RCB. Pain assessment in the patient unable to self-report: clinical practice recommendations in support of the ASPMN 2019 position statement. Pain Manag Nurs. 2019;20(5):404–417. doi:10.1016/j.pmn.2019.07.005. 31610992

[7] Georgiou E, Hadjibalassi M, Lambrinou E, Andreou P, Papathanassoglou ED. The impact of pain assessment on critically Ill patients’ outcomes: a systematic review. Biomed Res Int. 2015;2015:503830. doi:10.1155/2015/5038301-18. PMC4628961 26558273

[8] Shahid A, Sept BG, Moss SJ, Brundin-mather R, Krewulak KD, Soo A, Leigh JP, Gélinas C, Fiest KM, Stelfox HT, et al. Feasibility and acceptability of a patient- and family-oriented approach to pain assessment in the intensive care unit. Nurs Crit Care. 2026;31(3):e70459. doi:10.1111/nicc.70459. PMC13006934 41867117

[9] Azevedo-Santos IF, DeSantana JM. Pain measurement techniques: spotlight on mechanically ventilated patients. J Pain Res. 2018;11:2969–2980. doi:10.2147/JPR.S151169. PMC6255280 30538536

[10] Devlin JW, Skrobik Y, Gelinas C, Needham DM, Slooter AJ, Pandharipande PP, Watson PL, Weinhouse GL, Nunnally, ME, Rochwerg B, et al. Clinical practice guidelines for the prevention and management of pain, agitation/sedation, delirium, immobility, and sleep disruption in adult patients in the ICU. Crit Care Med. 2018;46(9):e825–e873. doi:10.1097/CCM.0000000000003299. 30113379

[11] Gelinas C, Joffe AM, Szumita PM, Hagg-Rickert K, Ouellet JF, Bérubé M, Arbour C. A psychometric analysis update of behavioral pain assessment tools for noncommunicative, critically ill adults. AACN Adv Crit Care. 2019;30(4):365–387. doi:10.4037/aacnacc2019952. 31951666

[12] Khanna P, Pandey RK, Chandralekha C, Sharma A, Pangasa N. Comparison between critical-care pain observation tool and physiologic indicators for pain assessment in the critically ill, mechanically ventilated adult patients. Saudi J Anaesth. 2018;12(3):384–388. doi:10.4103/sja.SJA_642_17. PMC6044155 30100835

[13] Shahid A, Sept BG, Longmore S, Owen VS, Moss SJ, Soo A, Fiest KM, Gélinas C, Stelfox HT. Development and preclinical testing of the critical care pain observation tool for family caregiver use (CPOT-Fam). Health Sci Rep. 2023;6(1):e986. doi:10.1002/hsr2.986. PMC9732740 36514328

[14] SB SA, Owen VS, Johnstone C, Paramalingam R, Moss SJ, Brundin-Mather R, Krewulak KD, Soo A, Parsons-Leigh J, Gélinas C, et al. 2023. Preliminary clinical testing to inform development of the critical care pain observation tool for families (CPOT-Fam). Canadian J of Pain. Accepted author version posted online doi:10.1080/24740527.2023.2235399 PMC10503446 37719471

[15] Shahid A, Owen V, Sept B, Longmore S, Soo A, Brundin-Mather R, Krewulak KD, Moss SJ, Plotnikoff KM, Gélinas C, et al. Study protocol: development and pilot testing of the critical care pain observation tool for families (CPOT-Fam). Pilot and Feasibility Studies. 2022;8(1). doi:10.1186/s40814-022-01102-3. PMC9287531 35842680

[16] Echegaray-Benites C, Kapoustina O, Gelinas C. Validation of the use of the critical-care pain observation tool (CPOT) with brain surgery patients in the neurosurgical intensive care unit. Intensive Crit Care Nurs. 2014;30(5):257–265. doi:10.1016/j.iccn.2014.04.002. 24836539

[17] Mohand-Saïd S, Lalonde MR, Boitor M, Gélinas C. Family members’ experiences with observing pain behaviors using the critical-care pain observation tool. Pain Manage Nurs. 2019;20(5):455–461. doi:10.1016/j.pmn.2018.11.001. 31109880

[18] Kotfis K, Zegan-Baranska M, Szydlowski L, Zukowski M, Ely EW. Methods of pain assessment in adult intensive care unit patients - polish version of the CPOT (critical care pain observation tool) and BPS (behavioral pain scale). Anaesthesiol Intensive Ther. 2017;49(1):66–72. doi:10.5603/AIT.2017.0010. 28362033

[19] Gelinas C, Fillion L, Puntillo KA, Viens C, Fortier M. Validation of the critical-care pain observation tool in adult patients. Am J Crit Care. 2006;15(4):420–427. doi:10.4037/ajcc2006.15.4.420. 16823021

[20] Frandsen JB, O’reilly Poulsen KS, Laerkner E, Stroem T. Validation of the Danish version of the critical care pain observation tool. Acta Anaesthesiol Scand. 2016;60(9):1314–1322. doi:10.1111/aas.12770. 27468726

[21] Pudas-Tahka SM, Axelin A, Aantaa R, Lund V, Salantera S. Translation and cultural adaptation of an objective pain assessment tool for Finnish ICU patients. Scand J Caring Sci. 2014;28(4):885–894. doi:10.1111/scs.12103. 24304287

[22] Kiesewetter I, Bartels U, Bauer A, Schneider G, Pilge S. The german version of the critical-care pain observation tool for critically ill adults: a prospective validation study. Anaesthesist. 2019;68(12):836–842. Die Deutsche Version des Critical-Care-Pain-Observation-Tools: Eine prospektive Validierungsstudie. doi:10.1007/s00101-019-00694-5. 31748831

[23] Sulla F, De Souza Ramos N, Terzi N, Trenta T, Uneddu M, Zaldivar Cruces MA, Sarli L. Validation of the Italian version of the Critical Pain Observation Tool in brain-injured critically ill adults. Acta Biomed. 2017;88(5S):48–54. doi:10.23750/abm.v88i5-S.6858. PMC6357580 29189705

[24] Kwak EM, Oh H. Validation of a Korean translated version of the critical care pain observation tool (CPOT) for ICU patients. J Korean Acad Nurs. 2012;42(1):76–84. doi:10.4040/jkan.2012.42.1.76. 22410604

[25] Hsiung NH, Yang Y, Lee MS, Dalal K, Smith GD. Translation, adaptation, and validation of the behavioral pain scale and the critical-care pain observational tools in Taiwan. J Pain Res. 2016;9:661–669. doi:10.2147/JPR.S91036. PMC5029847 27695360

[26] Kotfis K, Zegan-Baranska M, Strzelbicka M, Safranow K, Żukowski M, Ely EW. Validation of the Polish version of the Critical Care Pain Observation Tool (CPOT) to assess pain intensity in adult, intubated intensive care unit patients: the POL-CPOT study. Arch Med Sci. 2018;14(4):880–889. doi:10.5114/aoms.2017.69752. PMC6040120 30002708

[27] Marques R, Araujo F, Fernandes M, Freitas J, Dixe MA, Gelinas C. Validation testing of the european portuguese Critical-Care Pain Observation Tool. *Healthcare (Basel)*. 2022;10(6). 10.3390/healthcare10061075 PMC9222682 35742126

[28] Arroyo-Novoa CM, Figueroa-Ramos MI, Puntillo KA, Gelinas C. Translation into Spanish and cultural adaptation of the critical-care pain observation tool. Am J Crit Care. 2020;29(3):226–232. doi:10.4037/ajcc2020763. PMC7307381 32355973

[29] Aktas YY, Karabulut N. A Turkish version of the Critical-Care Pain Observation Tool: reliability and validity assessment. J Perianesth Nurs. 2017;32(4):341–351. doi:10.1016/j.jopan.2015.12.015. 28739066

[30] Joffe AM, McNulty B, Boitor M, Marsh R, Gelinas C. Validation of the Critical-Care Pain Observation Tool in brain-injured critically ill adults. J Crit Care. 2016;36:76–80. doi:10.1016/j.jcrc.2016.05.011. 27546751

[31] Linde SM, Badger JM, Machan JT, Beaudry J, Brucker A, Martin K, Opaluch-Bushy NB, Navedo Roy RD. Reevaluation of the critical-care pain observation tool in intubated adults after cardiac surgery. Am J Crit Care. 2013;22(6):491–497. doi:10.4037/ajcc2013700. 24186820

[32] Rijkenberg S, Stilma W, Bosman RJ, van der Meer NJ, van der Voort PHJ. Pain measurement in mechanically ventilated patients after cardiac surgery: comparison of the Behavioral Pain Scale (BPS) and the Critical-Care Pain Observation Tool (CPOT). J Cardiothorac Vasc Anesth. 2017;31(4):1227–1234. doi:10.1053/j.jvca.2017.03.013. 28800982

[33] Kanji S, MacPhee H, Singh A, Johanson C, Fairbairn J, Lloyd T, MacLean R, Rosenberg E. Validation of the Critical Care Pain Observation Tool in critically Ill patients with delirium: a prospective cohort study. Crit Care Med. 2016;44(5):943–947. doi:10.1097/CCM.0000000000001522. 26783859

[34] Fedele S, Strasser S, Roulin MJ. Validation of the critical care oain observational tool in palliative care. Pain Manag Nurs. 2020;21(4):360–364. doi:10.1016/j.pmn.2019.12.003. 32113802

[35] Tao H, Galagarza SR. P-CPOT: an adaptation of the Critical-Care Pain Observation Tool for pediatric intensive care unit patients. Pain Manag Nurs. 2020;21(2):172–178. doi:10.1016/j.pmn.2019.07.012. 31506237

[36] MMJ L, Ocay DD, Larche CL, Vickers K, Saran N, Ouellet JA, Gélinas C, Ferland CE. Validation of the Critical-Care Pain Observation Tool (CPOT) in pediatric patients undergoing orthopedic surgery. Can J Pain. 2023;7(1):2156332. doi:10.1080/24740527.2022.2156332. PMC9980602 36874228

[37] Viera AJ, Garrett JM. Understanding interobserver agreement: the kappa statistic. Fam Med. 2005;37(5):360–363. 15883903

[38] Mukaka MM. Statistics corner: a guide to appropriate use of correlation coefficient in medical research. Malawi Med J. 2012;24(3):69–71. PMC3576830 23638278

[39] Faria-Schutzer DB, Surita FG, Alves VLP, Bastos RA, Campos CJG, Turato ER. Seven steps for qualitative treatment in health research: the clinical-qualitative content analysis. Cien Saude Colet. 2021;26(1):265–274. doi:10.1590/1413-81232020261.07622019. 33533847

[40] Gohil S, Vuik S, Darzi A. Sentiment analysis of health care tweets: review of the methods used. JMIR Public Health Surveill. 2018;4(2):e43. doi:10.2196/publichealth.5789. PMC5938573 29685871

[41] Hajian-Tilaki K. Sample size estimation in diagnostic test studies of biomedical informatics. J Biomed Inform. 2014;48:193–204. doi:10.1016/j.jbi.2014.02.013. 24582925

[42] Fiest KM, Krewulak KD, Sept BG, Davidson JE, Ely EW, Lee CH, Soo A, Stelfox HT. A pilot randomized controlled trial assessing the feasibility and acceptability of family-partnered delirium prevention, detection, and management in critically Ill adults: the Activating Family Caregivers in the Identification Prevention and Management of Delirium (ACTIVATE) Study. Crit Care Explor. 2025;7(9):e1287. doi:10.1097/CCE.0000000000001287. PMC12377300 40844707

[43] Weiss DJ, Wang C, Suen KY, Basford J, Cheville A. Can proxy ratings supplement patient report to assess functional domains among hospitalized patients? Arch Phys Med Rehabil. 2022;103(5S):S34–S42e4. doi:10.1016/j.apmr.2021.08.024. PMC9018891 34678294

[44] Kroenke K, Stump TE, Monahan PO. Agreement between older adult patient and caregiver proxy symptom reports. J Patient Rep Outcomes. 2022;6(1):50. doi:10.1186/s41687-022-00457-8. PMC9107556 35567663

[45] Desbiens NA, Mueller-Rizner N. How well do surrogates assess the pain of seriously ill patients? Crit Care Med. 2000;28(5):1347–1352. doi:10.1097/00003246-200005000-00015. 10834677

[46] Price AM, McAndrew NS, Thaqi Q, Kirk M, Brysiewicz P, Eggenberger S, Naef R. Factors influencing critical care nurses’ family engagement practices: an international perspective. Nurs Crit Care. 2022;doi:10.1111/nicc.12824 35831205

[47] Marra A, Ely EW, Pandharipande PP, Patel MB. The ABCDEF bundle in critical care. Crit Care Clin. 2017;33(2):225–243. doi:10.1016/j.ccc.2016.12.005. PMC5351776 28284292

[48] Shahid A, Johnstone C, Sept BG, Kupsch S, Pryznyk J, Elton-LaCasse C, Everson J, Soo A, Jaworska N, Fiest KM, et al. Family-led coaching of patients during weaning from sedation and mechanical ventilation in the ICU. Respir Care. 2025;70(2):134–142. doi:10.4187/respcare.11780. 39379158

[49] Parsons Leigh J, Mizen SJ, Moss SJ, Rieger KL, McCallum A, Dodek PM, Stelfox HT, Fiest KM. A qualitative descriptive study of the impact of the COVID-19 pandemic on staff in a Canadian intensive care unit [Une etude qualitative descriptive de l’impact de la pandemie de COVID-19 sur le personnel d’une unite de soins intensifs canadienne]. Can J Anaesth. 2023;70(3):384–394. 10.1007/s12630-022-02377-z. PMC9831684 36627462

[50] Julious SA. Sample size of 12 per group rule of thumb for a pilot study. Pharm Stat. 2005;4(4):287–291. doi:10.1002/pst.185.

[51] Zhang L, Losin EAR, Ashar YK, Koban L, Wager TD. Gender biases in estimation of others’ pain. J Pain. 2021;22(9):1048–1059. doi:10.1016/j.jpain.2021.03.001. PMC8827218 33684539

